# Supplementary material for: Forest habitat parameters influence abundance and diversity of cadaver-visiting dung beetles in Central Europe
Source: R Soc Open Sci. 2020 Mar 4;7(3):191722. doi: 10.1098/rsos.191722 (PMC7137943; doi:10.1098/rsos.191722)
Supplement: Environmental variables and results of quasi-Poisson-GLMs and Gaussian-GLMs [file rsos191722supp1.doc]

**Table 1.**Environmental variables used in this study, obtained from the BExIS platform (Biodiversity Exploratories Information System, https://www.bexis.uni-jena.de); modified after [1].

| **Variable** | **Variable type** | **Categories or description of variable** | **[unit]** | **Data source information** |
| --- | --- | --- | --- | --- |
| location | categorial | Schwäbische Alb (AEW), Hainich-Dün (HEW), Schorfheide-Chorin (SEW) | - | [2] |
| management system | categorial | age-class (AC), extensively_managed, selection_system, unmanaged | - | id: 17706, version: 1.2.3; [3] owner: Schall & Ammer |
| main tree species | categorial | Beech, Spruce, Pine | - | id: 10580 , version: 2.7.6; [4] owner: Nieschulze & Schulze |
| mean ambient temperature | continuous | mean ambient temperature in the immediate vicinity of an exposed cadaver (averages from half-hourly recorded values of data loggers within wire cages) | °C | [2] |
| air humidity | continuous | relative air humidity at 2 m above ground | % | id: 19007, version: 1.0.5; [5] owner: Wöllauer, Hänsel, Nauss, Forteva |
| stand age | continuous | age in year 2012 of main tree species | years | id: 17486, version: 1.1.3; [6] owner: Schall & Ammer |
| stand density MTS | continuous | stand density of main tree species (MTS) | trees/ha | id: 17687, version: 1.4.5; [7] owner: Schall & Ammer |
| crown closure | categorial | compact, closed, loose_sparse, spacious_interrupted | - | id: 10580 , version: 2.7.6; [4] owner: Nieschulze & Schulze |
| forest understory | continuous | understory up to 2 m above the ground | proportion | id: 17066, version: 1.1.3; [8] owner: Schall & Ammer |
| dbh standard deviation | continuous | standard deviation of dbh (diameter in breast height) | cm | id: 17687, version: 1.4.5; [7] owner: Schall & Ammer |
| shannon total vascular plants | continuous | Shannon diversity index for all vascular plants | - | id: 16806, version: 1.2.2; [9] owner: Grassein & Fischer |
| simpson total vascular plants | continuous | Simpson diversity index for all vascular plants | - | id: 16806, version: 1.2.2; [9] owner: Grassein & Fischer |
| soil type | categorial | Albeluvisol, Cambisol, Leptosol, Luvisol, Stagnosol | - | id: 10580 , version: 2.7.6; [4] owner: Nieschulze & Schulze |
| soil temperature | continuous | soil temperature at 10 cm below surface | °C | id: 19007, version: 1.0.5; [5] owner: Wöllauer, Hänsel, Nauss, Forteva |
| soil moisture | continuous | soil moisture at 10 cm below surface | % | id: 19007, version: 1.0.5; [5] owner: Wöllauer, Hänsel, Nauss, Forteva |
| mineral soil pH | continuous | pH-measurement in the mineral soil  (0 - 10 cm) | - | id: 19067, version: 4.1.2; [10] owner: Schöning, Klötzing, Schäfer, Gan, Schrumpf, Trumbore |
| bulk density | continuous | bulk density for the upper 10 cm of the mineral soil | g/cm^3^ | id: 17086, version: 1.1.5; [11] owner: Schöning, Solly, Klötzing, Trumbore, Schrumpf |
| fine sand | continuous | percentage of fine sand (particle size: 0.063 - 0.2 mm) in soil samples | g/kg soil | id: 14686, version: 1.9.6; [12] owner: Schöning, Solly, Klötzing, Trumbore, Schrumpf |
| fine silt | continuous | percentage of fine silt (particle size: 0.002 - 0.0063 mm) in soil samples | g/kg soil | id: 14686, version: 1.9.6; [12] owner: Schöning, Solly, Klötzing, Trumbore, Schrumpf |
| clay | continuous | percentage of clay (particle size: < 0.002 mm) in soil samples | g/kg soil | id: 14686, version: 1.9.6; [12] owner: Schöning, Solly, Klötzing, Trumbore, Schrumpf |
| forest utilization intensity SMI | continuous | forest utilization intensity index (from 0 to 1) 0 = undisturbed 1 = disturbed | - | id: 17746 , version: 1.2.2; [13] owner: Schall & Ammer |

**Table 2.**Variable selection based on random forest. The random forest method was used in order to determine which habitat variables were more important to predict the total abundance of **a** all scarabaeoid beetle taxa (Scarabaeoidea) and of the single scarabaeoid species **b** *Anoplotrupes stercorosus* and **c** *Trypocopris vernalis*. This is performed by running regression trees at high numbers (here number of runs = 10^5^, [1]). This method [14,15] works even for highly correlated variables, allowing similar important scores to each. *A priori*, for quasi-Poisson-GLMs, we chose the seven most important habitat variables (the best third of all variables) for a subsequent stepwise selection in both directions (backwards and forwards, starting with the full model, after [1,16]).

|  | ***(a)* Total abundance Scarabaeoidea** |  |
| --- | --- | --- |
|  | **Variable** | **Increment of Node Purity** |
| 1 | mean ambient temperature | 366160.36 |
| 2 | soil moisture | 277015.80 |
| 3 | fine sand | 260748.09 |
| 4 | mineral soil pH | 190508.32 |
| 5 | soil temperature | 173833.21 |
| 6 | clay | 170273.40 |
| 7 | fine silt | 143469.95 |
| excluded | location | 122110.08 |
| excluded | forest understory | 92830.51 |
| excluded | bulk density | 89867.66 |
| excluded | stand density MTS | 81911.87 |
| excluded | stand age | 73756.09 |
| excluded | dbh standard deviation | 58238.82 |
| excluded | Shannon total vascular plants | 53513.04 |
| excluded | air humidity | 50247.96 |
| excluded | Simpson total vascular plants | 48273.58 |
| excluded | main tree species | 15353.11 |
| excluded | crown closure | 7578.05 |
| excluded | soil type | 5609.38 |
| excluded | management system | 2724.91 |
|  | ***(b)* Total abundance *A. stercorosus*** |  |
| 1 | mean ambient temperature | 312969.70 |
| 2 | fine sand | 221355.79 |
| 3 | soil moisture | 211704.88 |
| 4 | mineral soil pH | 201516.22 |
| 5 | soil temperature | 155963.50 |
| 6 | clay | 149542.86 |
| 7 | fine silt | 130450.74 |
| excluded | location | 104781.94 |
| excluded | bulk density | 93975.09 |
| excluded | forest understory | 78276.38 |
| excluded | stand density MTS | 67124.51 |
| excluded | stand age | 61908.99 |
| excluded | Shannon total vascular plants | 59980.01 |
| excluded | Simpson total vascular plants | 59323.49 |
| excluded | dbh standard deviation | 53848.77 |
| excluded | air humidity | 51113.82 |
| excluded | crown closure | 8475.09 |
| excluded | main tree species | 7151.73 |
| excluded | soil type | 5083.49 |
| excluded | management system | 2189.58 |
|  | ***(c)* Total abundance *T. vernalis*** (Schorfheide-Chorin) | |
| 1 | Shannon total vascular plants | 6236.79 |
| 2 | Simpson total vascular plants | 6153.90 |
| 3 | fine sand | 2757.74 |
| 4 | stand age | 2016.01 |
| 5 | stand density MTS | 1541.28 |
| 6 | dbh standard deviation | 1100.62 |
| 7 | soil moisture | 1093.96 |
| excluded | forest understory | 1084.16 |
| excluded | mean ambient temperature | 1062.08 |
| excluded | clay | 423.80 |
| excluded | soil temperature | 417.96 |
| excluded | mineral soil pH | 240.83 |
| excluded | main tree species | 178.59 |
| excluded | air humidity | 169.12 |
| excluded | bulk density | 157.89 |
| excluded | fine silt | 130.36 |
| excluded | crown closure | 53.92 |
| excluded | management system | 0.18 |
| excluded | soil type | 0.01 |

**Table 3.**Results of quasi-Poisson-GLMs comparing total abundance of **a** all scarabaeoid dung beetle taxa ~~(Scarabaeoidea)~~ and of **b** the single geotrupid species *Anoplotrupes stercorosus* in the different forest types in three regions. **c** For the geotrupid species *Trypocopris vernalis*, results of quasi-Poisson-GLMs are shown comparing its total abundance in the different forest types in the Schorfheide-Chorin region. Bold text indicates significant effects (*α=*0.05). Important environmental variables were fitted first, according to their importance (electronic supplementary material, table 2). For GLMs, stepwise model simplification (based on quasi-Akaike information criterion QAIC) was performed. Letters indicate the sequence of variable exclusion from the full GLM model, (starting with ‘a’). Letters combined with ‘+’ indicate when a previously excluded variable was included into the GLM model once again [1].

|  | Quasi-Poisson-GLM | |
| --- | --- | --- |
|  | *F* | *p* |
| *(a) Abundance of all dung beetle species* |  |  |
| **mean ambient temperature** | **91.71 (b +)** | **< 0.001** |
| fine sand | 3.31 | 0.075 |
| soil moisture | 3.17 | 0.081 |
| mineral soil pH | 2.12 | 0.152 |
| clay | 0.38 | 0.539 |
| soil temperature | 0.33 | 0.566 |
| fine silt | A |  |
| *(b) Abundance of geotrupid species* A. stercorosus |  |  |
| **mean ambient temperature** | **83.19** | **< 0.001** |
| **fine sand** | **4.29** | **0.044** |
| mineral soil pH | 2.36 | 0.131 |
| soil moisture | 1.19 | 0.282 |
| clay | 0.49 | 0.487 |
| soil temperature | 0.21 (b +) | 0.652 |
| fine silt | A |  |
| *(c)* *Abundance of geotrupid species* T. vernalis |  |  |
| **Shannon total vascular plants** | **230.96** | **< 0.001** |
| **stand age** | **47.58** | **< 0.001** |
| **fine sand** | **22.52** | **< 0.001** |
| **Simpson total vascular plants** | **10.88** | **0.007** |
| **stand density MTS** | **8.27** | **0.015** |
| soil moisture | 3.62 | 0.084 |
| dbh standard deviation | A |  |

**Table 4.**Variable selection based on random forest. The random forest method was used in order to determine which habitat variables were more important to predict the species richness of the taxon Scarabaeoidea limited by trapped beetle taxa *Anoplotrupes stercorosus*, *Trypocopris vernalis*, *Onthophagus coenobita*, *Onthophagus similis*, *Aphodius rufipes*, *Aphodius sticticus*, *Onthophagus fracticornis* and *Onthophagus ovatus*. This is performed by running regression trees at high numbers (here number of runs = 10^5^, [1]). This method [14,15] works even for highly correlated variables, allowing similar important scores to each. *A priori*, we chose the seven most important habitat variables (the best third of all 20 variables) for a subsequent stepwise selection in both directions (backwards and forwards, starting with the full model, after [1,16]).

|  | **Species richness Scarabaeoidea** |  |
| --- | --- | --- |
|  | **Variable** | **Increment of Node Purity** |
| 1 | soil moisture | 6.76 |
| 2 | mineral soil pH | 6.68 |
| 3 | mean ambient temperature | 4.94 |
| 4 | fine sand | 3.11 |
| 5 | clay | 2.39 |
| 6 | forest understory | 2.23 |
| 7 | fine silt | 2.17 |
| excluded | location | 1.67 |
| excluded | soil temperature | 1.33 |
| excluded | main tree species | 1.31 |
| excluded | stand age | 0.80 |
| excluded | bulk density | 0.64 |
| excluded | stand density MTS | 0.60 |
| excluded | dbh standard deviation | 0.55 |
| excluded | Shannon total vascular plants | 0.54 |
| excluded | Simpson total vascular plants | 0.43 |
| excluded | air humidity | 0.42 |
| excluded | soil type | 0.16 |
| excluded | crown closure | 0.12 |
| excluded | management system | 0.04 |

**Table 5.**
Results of quasi-Poisson-GLMs comparing species richness of dung beetles (including the beetle taxa *Anoplotrupes stercorosus*, *Trypocopris vernalis*, *Onthophagus coenobita*, *Onthophagus similis*, *Aphodius rufipes*, *Aphodius sticticus*, *Onthophagus fracticornis* and *Onthophagus ovatus*) in the different forest types in three regions. Bold text indicates significant effects (*α=*0.05). Important environmental variables (electronic supplementary material, table 4) were fitted first, according to their importance. Stepwise model simplification (based on quasi-Akaike information criterion QAIC) was performed. Letters indicate the sequence of variable exclusion from the full GLM model (starting with ‘a’, [after 1]).

|  | Quasi-Poisson-GLM | |
| --- | --- | --- |
|  | *F* | *p* |
| *Dung beetle species richness* |  |  |
| **soil moisture** | **98.19** | **< 0.001** |
| **forest understory** | **9.65** | **0.003** |
| fine sand | 3.58 | 0.065 |
| fine silt | 2.73 | 0.105 |
| clay | A |  |
| mean ambient temperature | b |  |
| mineral soil pH | c |  |

**Table 6.**

Variable selection based on random forest. The random forest method was used in order to determine which habitat variables were more important to predict the Shannon’s diversity and the Simpson’s dominance of the Scarabaeoidea taxon. This is performed by running regression trees at high numbers (here number of runs = 10^5^, [1]). This method [14,15] works even for highly correlated variables, allowing similar important scores to each. *A priori*, for Gaussian-GLMs (link = “identity”, after [17]), we chose the seven most important habitat variables (the best third of all variables) for a subsequent stepwise selection in both directions (backwards and forwards, starting with the full model, after [1,16]).

|  | **Shannon’s diversity Scarabaeoidea** |  |
| --- | --- | --- |
|  | **Variable** | **Increment of Node Purity** |
| 1 | Shannon total vascular plants | 0.096 |
| 2 | Simpson total vascular plants | 0.077 |
| 3 | stand age | 0.056 |
| 4 | stand density MTS | 0.046 |
| 5 | dbh standard deviation | 0.045 |
| 6 | forest understory | 0.043 |
| 7 | mean ambient temperature | 0.038 |
| excluded | soil moisture | 0.032 |
| excluded | main tree species | 0.015 |
| excluded | fine sand | 0.014 |
| excluded | soil temperature | 0.011 |
| excluded | crown closure | 0.009 |
| excluded | clay | 0.008 |
| excluded | mineral soil pH | 0.007 |
| excluded | fine silt | 0.006 |
| excluded | air humidity | 0.005 |
| excluded | bulk density | 0.004 |
| excluded | soil type | 0.00013 |
| excluded | management system | 0.00010 |
|  | **Simpson’s dominance Scarabaeoidea** |  |
| 1 | Shannon total vascular plants | 0.0981 |
| 2 | Simpson total vascular plants | 0.0862 |
| 3 | stand age | 0.0487 |
| 4 | mean ambient temperature | 0.0475 |
| 5 | soil moisture | 0.0467 |
| 6 | stand density MTS | 0.0370 |
| 7 | fine sand | 0.0295 |
| excluded | forest understory | 0.0269 |
| excluded | dbh standard deviation | 0.0256 |
| excluded | soil temperature | 0.0084 |
| excluded | clay | 0.0072 |
| excluded | main tree species | 0.0069 |
| excluded | crown closure | 0.0047 |
| excluded | mineral soil pH | 0.0046 |
| excluded | fine silt | 0.0043 |
| excluded | air humidity | 0.0041 |
| excluded | bulk density | 0.0027 |
| excluded | management system | 0.00003 |
| excluded | soil type | 0.00002 |

**Table 7.**

Results of Gaussian-GLMs (link = “identity”) comparing Shannon’s diversity and Simpson’s dominance of dung beetles in the different forest types in the Schorfheide-Chorin region. Bold text indicates significant effects (*α=*0.05). Important environmental variables (electronic supplementary material, table 6) were fitted first, according to their importance. Stepwise model simplification (based on Akaike information criterion AIC) was performed. Letters indicate the sequence of variable exclusion from the full GLM model (starting with ‘a’, after [after 1]).

|  | Gaussian-GLM | |
| --- | --- | --- |
|  | *F* | *p* |
| *(a) Dung beetle Shannon’s diversity* |  |  |
| **Shannon total vascular plants** | **23.94** | **< 0.001** |
| dbh standard deviation | 3.99 | 0.069 |
| mean ambient temperature | 3.03 | 0.107 |
| Simpson total vascular plants | 1.97 | 0.186 |
| stand density MTS | 0.02 | 0.901 |
| forest understory | a |  |
| stand age | b |  |
| *(b) Dung beetle Simpson’s dominance* |  |  |
| **Shannon total vascular plants** | **15.95** | **0.0012** |
| **fine sand** | **6.44** | **0.023** |
| mean ambient temperature | a |  |
| soil moisture | b |  |
| stand density MTS | c |  |
| stand age | d |  |
| Simpson total vascular plants | e |  |

REFERENCES

1. Lange M, Türke M, Pašalić E, Boch S, Hessenmöller D, Müller J, Prati D, Socher SA, Fischer M, Weisser WW, et al. 2014 Effects of forest management on ground-dwelling beetles (Coleoptera; Carabidae, Staphylinidae) in Central Europe are mainly mediated by changes in forest structure. *For. Ecol. Manage*. **329**, 166-176. (doi:10.1016/j.foreco.2014.06.012)
2. 10.5061/dryad.1ns1rn8ps
3. Schall P, Ammer C. 2018 New forest type classification of all forest EPs, 2008-2014. v1.2.5. *Biodiversity Exploratories Information System. Dataset*. <https://www.bexis.uni-jena.de/PublicData/PublicData.aspx?DatasetId=17706>
4. Ostrowski A, Nieschulze J, Schulze ED, Fischer M, Ayasse M, Weisser W, König-Ries B. 2016 BE Project - Basic Information of Field Plots. v1.1.4. *Biodiversity Exploratories Information System*. Dataset. <https://www.bexis.uni-jena.de/PublicData/PublicData.aspx?DatasetId=1000>
5. Hänsel F, Forteva S, Wöllauer S, Nauss T. 2019 Öffentlich verfügbare Klimadaten der Exploratorien / Open Climate Data of the Exploratories Project. v1.0.11. *Biodiversity Exploratories Information System*. Dataset. <https://www.bexis.uni-jena.de/PublicData/PublicData.aspx?DatasetId=24766>
6. Schall P, Ammer C. 2018 Stand age of all forest EPs, 2012. v1.1.5. *Biodiversity Exploratories Information System*. Dataset. <https://www.bexis.uni-jena.de/PublicData/PublicData.aspx?DatasetId=17486>
7. Schall P, Ammer C. 2016 Forest EP stand structure and composition. v1.4.5. *Biodiversity Exploratories Information System*. Dataset. <https://www.bexis.uni-jena.de/PublicData/PublicData.aspx?DatasetId=17687>
8. Schall P, Ammer C. 2016 EP-summary of Lidar derived forest structure parameter. v1.1.4. *Biodiversity Exploratories Information System*. Dataset. <https://www.bexis.uni-jena.de/PublicData/PublicData.aspx?DatasetId=17066>
9. Grassein F, Fischer M. 2018 Vegetation Records for Forest EPs in 2012, Header Data without Species Identities. v1.2.5*. Biodiversity Exploratories Information System*. Dataset. <https://www.bexis.uni-jena.de/PublicData/PublicData.aspx?DatasetId=16806>
10. Schöning I, Solly E, Klötzing T, Schrumpf M. 2019 Mineral soil pH values of all experimental plots (EP) of the Biodiversity Exploratories project from 2011, Soil (core project). v1.10.28. *Biodiversity Exploratories Information System*. Dataset. <https://doi.org/10.25829/bexis.14447-1.10.28>
11. Schöning I, Solly E, Klötzing T, Trumbore S, Schrumpf M. 2018 MinSoil 2011 - Soil Bulk Density and Carbon and Nitrogen stocks. v1.2.11. *Biodiversity Exploratories Information System*. Dataset. <https://www.bexis.uni-jena.de/PublicData/PublicData.aspx?DatasetId=17086>
12. Herold N, Schrumpf M. 2016 Soil Texture Mixed Samples VIP Plots, 2008. v1.1.6. *Biodiversity Exploratories Information System*. Dataset. <https://www.bexis.uni-jena.de/PublicData/PublicData.aspx?DatasetId=6661>
13. Schall P, Ammer C. 2018 SMI - Silvicultural management intensity index on all forest EPs, 2008-2014. v1.2.4. *Biodiversity Exploratories Information System*. Dataset. <https://www.bexis.uni-jena.de/PublicData/PublicData.aspx?DatasetId=17746>
14. Breiman L. 2001 Random forests. *Mach. Learn*. **45**, 5-32. (doi:10.1023/A:101093340)
15. Prasad AM, Iverson LR, Liaw A. 2006 Newer classification and regression tree techniques: Bagging and random forests for ecological prediction. *Ecosystems* **9**, 181-199. (doi:10.1007/s10021-005-0054-1)
16. Loranger J, Meyer ST, Shipley B, Kattge J, Loranger H, Roscher C, Weisser WW. 2012 Predicting invertebrate herbivory from plant traits: evidence from 51 grassland species in experimental monocultures. *Ecology* **93**, 2674-2682. (doi:10.1890/12-0328.1)
17. Roiz D, Ruiz S, Soriguer R, Figuerola J. 2015 Landscape effects on the presence, abundance and diversity of mosquitoes in Mediterranean wetlands. *PLoS ONE* **10**, e0128112. (doi:10.1371/journal.pone.0128112)
